# Long-Term Outcome of* En Bloc* Extensive Resection of the Penis and Prepuce Associated with a Permanent Perineal Urethrostomy in a Gelding Affected by Squamous Cell Carcinoma

**DOI:** 10.1155/2016/6989450

**Published:** 2016-09-27

**Authors:** Paola Straticò, Vincenzo Varasano, Gianluca Celani, Riccardo Suriano, Lucio Petrizzi

**Affiliations:** Faculty of Veterinary Medicine, University of Teramo, OVUD, Località Piano D'Accio, 64100 Teramo, Italy

## Abstract

A 15-year-old gelding was referred for a florid, cauliflower-like ulcerated mass, enclosing penis and prepuce together with penile urethra showing a malodorous purulent and blood-stained discharge and larvae infestation.* En bloc* extensive resection of the penis and prepuce, without penile retroversion or pexy to ventral abdomen associated with a permanent perineal urethrostomy, was performed. Histology of the mass revealed a squamous cell carcinoma of penis and prepuce. The surgical technique that was adopted is a modified version of that already described that allows a more proximal resection of the penile body and is a valid option for treating advanced SCC lesions involving the penis. Early postsurgical complications (mild strangury, haemorrhage from the urethrostomy site and its partial dehiscence, and infection of the abdominal wound) were managed with a medical treatment and resolved within 5 to 12 days. Three years after surgery the horse is in good body condition and does not show any sign of recurrence or disorders related to the surgery.

## 1. Introduction

Penile and preputial tumours are among the most common neoplasms in the horse, accounting for 6–10% of all neoplastic disorders in this species [1], with squamous cell carcinomas (SCC) being the most common [2]. It may arise* de novo* or from a malignant transformation of a squamous papilloma [2, 3]. It commonly occurs on the glans and internal lamina of the prepuce. It is locally invasive and has a low grade of malignancy [2, 4].

Suggested treatments of small and not complicated SCC of penis and prepuce are cryotherapy [5, 6] and chemotherapy [7, 8].

For extensive SCC surgical excision is recommended. Segmental posthectomy (reefing) and partial phallectomy [9–12] are indicated if only the distal portion of the penis is involved and the remaining free part of the penis can extend beyond the preputial orifice during urination. If penis, prepuce, and regional lymph nodes are extensively involved, surgical options are* en bloc* resection with or without penile retroversion [13–15] or penile transection just distal to a perineal urethrostomy [16, 17].

In a long-term follow-up study, 9 cases of SCC infiltrating the penile body were described with a survival rate of 55% (5/9) after penile amputation and urethrostomy [2]. Mair et al. described* en bloc* resection of penis and prepuce and superficial inguinal lymph nodes with penile retroversion in 4 horses affected by SCC with 100% survival rate in 1-year follow-up without recurrences [4]. Penile amputation and sheath ablation without penile retroversion were first described by Doles et al. [14] in 25 geldings, with a positive long-term outcome in 8 horses. In the case series by Archer and Edwards [15], 5 geldings undergoing* en bloc* resection of the penis for SCC had a positive long-term follow-up without recurrences; in one case urine scalding due to lateral deviation of the urinary flow was reported. Another retrospective study by Van den Top et al. [1] reported that* en bloc* resection of penis and prepuce was performed in 13 out of 77 horses affected by SCC with lymph node enlargement and extensive genital involvement. Eight horses were available for follow-up and only one suffered neoplastic recurrence within 18 months [1]. Wylie and Payne described a modified surgical technique consisting in subischial urethrostomy and penile amputation with preputial ablation, for the treatment of different severe pathologies (SCC, melanomas, chronic preputial discharge, and paraphimosis) in 15 horses. Median follow-up time was 25.1 months, survival rate 18 months after surgery was 65%, and 6 out of 15 patients were euthanized, 4 of which for reasons related to the procedure [18].

Short- and long-term complication associated with the surgical procedures consisted in cystitis, mild to severe haemorrhage [13–15], wound swelling and infection, urine scalding, second-intention healing of urethral stoma after dehiscence of the suture line, and recurrence of neoplasia [1].

This case report describes the successful treatment of a penile SCC in a gelding with* en bloc* extensive resection of penis and prepuce without penile retroversion, together with a permanent perineal urethrostomy, after a 3-year follow-up period, adding information about long-term clinical outcome after surgical treatment.

## 2. Case Presentation

A 15-year-old Argentinian Warmblood gelding was referred for treatment of a long-standing penile lesion affected by myiasis.

**Figure 1:**
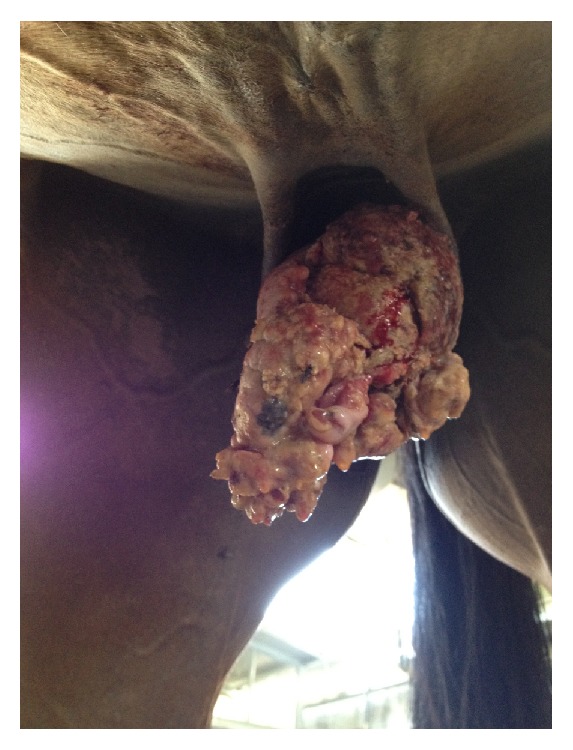
Macroscopic appearance of the penile mass at admission showing a florid, cauliflower-like ulcerated mass disrupting the normal anatomy of penis and prepuce.

At referral the main complaint was a florid, cauliflower-like ulcerated mass, measuring 10 cm in diameter that enclosed the* glans penis* together with penile urethra (Figure 1) with malodorous purulent and blood-stained discharge together with areas of necrosis. The mass was infested by* muscae* spp. larvae. Poor body condition score (BCS 4/9) and strangury were also present.

Once penis prolapse was obtained by intravenous administration with acepromazine 30 *μ*g/kg, palpation of the penis revealed a diffuse thickening of the penile shaft with multiple ulcerations involving the inner and outer laminae of the preputial fold. Due to the regional anatomical alterations related to the mass (phlegmon and oedema), inguinal lymph nodes were not palpable. For the same reason ultrasonographic examination of external genitalia and regional lymph nodes was not conclusive. Transrectal examination was unremarkable.

Urine analysis revealed the presence of leukocytes and nitrites, consistent with urinary tract inflammation. As the clinical condition of the horse and the primary lesion were severe, suggesting complete excision in any case, fine needle aspiration or excisional biopsies were not attempted; therefore surgical removal was combined with harvesting material for histopathological examination.

Based on clinical findings differential diagnoses were SCC of the penis and prepuce and/or habronemiasis. Due to the infiltrating pattern and the diffusion of the disorder and to the presence of multiple nonhealing lesions, leading, respectively, to difficulty at micturition and local infection, an* en bloc* resection of the penis and prepuce was decided; the eventuality of a penile retroversion or a permanent perineal urethrostomy was considered according to the intraoperatory findings and the degree of infiltration of the penis body [14]. Systemic NSAIDS (flunixin meglumine, 1.1. mg/kg i.v. q24 h) and antibiotic (sulfadiazine-trimethoprim, 30 kg/kg p.o. q24 h) therapies were initiated before surgery.

Surgery was performed under general anaesthesia with isoflurane with the horse in dorsal recumbence. For the* en bloc* resection, a fusiform 40 cm long skin incision starting at the umbilicus was made along the midline. It extended caudally encircling the preputial orifice. Blunt dissection of subcutaneous tissue was performed around the penis until the abdominal fascia was reached and the body of the penis was released from its anatomic and vascular connections. Haemorrhage was controlled with electrocauterization and ligation of the major vessels (dorsal penis arteries and veins). The dissection plane was extended to both the external inguinal rings and the external pudendal arteries and vein were ligated. The superficial inguinal lymph nodes appeared enlarged and therefore were excised. At palpation the penile body was extensively thickened suggesting amputation as proximal as possible: so penile retroversion was excluded and perineal urethrostomy was performed. To this aim, a tourniquet was applied proximally to the shaft of the penis and an extensive resection was performed close to the ischiatic arch, dividing the suspensory ligament of penis. The proximal stump showed a normal macroscopic appearance and was sutured with double transfixing sutures (2 USP absorbable multifilament) placed through the penile body in a dorsoventral direction, to obtain adequate haemostasis. Once urethral lumen was clearly visible a urinary catheter was placed. The outer perimeter of the tunica albuginea was apposed in a simple interrupted pattern with a 2/0 USP monofilament absorbable suture.

For permanent urethrostomy a 8 cm skin incision was created on the perineal raphe starting about 7-8 cm below the anus, ending at the level of the ischiatic arch. Blunt dissection of subcutis, penis retractor muscles, and bulbospongiosus muscles was achieved until the urethra was visualized. The penis retractor muscles were sutured to the subcutis in a simple continuous pattern, using a 0 USP monofilament absorbable material. Then the urethral wall was incised longitudinally for about 6 cm and the mucosa sutured to the skin in a simple interrupted pattern with a 2/0 USP monofilament nonabsorbable material.

**Figure 2:**
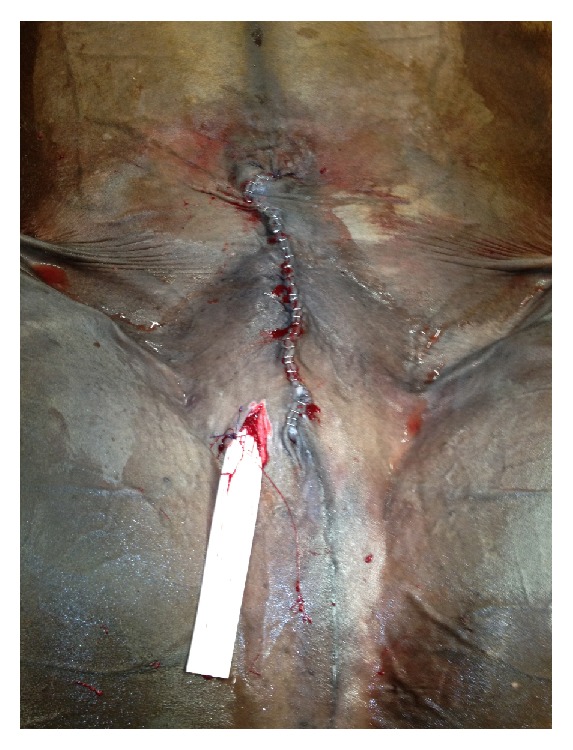
Abdominal wall suture and a multitubular drain applied at the scrotal region.

The subcutis of the abdominal wound was sutured with a 0 USP monofilament absorbable suture material in a simple continuous pattern and the skin was sutured with metallic staples. A multitubular drain was applied laterally to the midline to the abdominal wall suture at the scrotal region (Figure 2).

After recovery from anaesthesia the horse showed a moderate haemorrhage from the urethrostomy, which was particularly evident at the end of urination, leading after 48 h to a marked reduction of PCV (15%) and TP (4,5 g/dL). As the haemorrhage seemed to come from the corpus spongiosum penis and surgical haemostasis was not feasible, it was successfully managed by the administration of tranexamic acid (15 mg/kg i.v. q12 h) for 2 days. Oral support with vitamin B complex and folic and pantothenic acid was given for 10 days until PCV and TP reached, respectively, 30% and 6.8 g/dL.

Strangury but not pollakiuria was observed 3 days after surgery.

Three days after surgery, the horse showed a serosanguineous collection above the abdominal wound and a moderate purulent discharge from the skin incision that were managed with daily manual massage and local disinfection. Five days after surgery, the urinary catheter and the multitubular drain were removed without complications.

On day 12 standing surgical revision of the urethrostomy was required to remove some necrotic urethral mucosa leading to a partial dehiscence of the wound.

Histopathology of the lesions confirmed the presumptive diagnosis of SCC of the penis and prepuce invading the subcutaneous tissue, not the albuginea. Lymphocytic and neutrophilic infiltration of the corpus cavernosum suggested chronic balanitis; moderate subcutaneous eosinophilic infiltration was also detected but no evidence of* Habronema* spp. infestation was found. The margins of the excised tissues were free of neoplastic cells, and the regional lymph nodes were inflamed but not affected by the neoplastic process.

After skin staples removal 14 days after surgery, the horse was discharged. Urination was unremarkable from the perineal stoma. No urine scalding or subcutaneous infiltration could be observed. PCV and TP values were back to preoperatory values.

A clinical follow-up performed 2 months after surgery revealed reduction of the urethrostomy to a diameter of about 1.5/2 cm. Anyhow urination was unaffected with a mare attitude and no urine scalding was detected.

**Figure 3:**
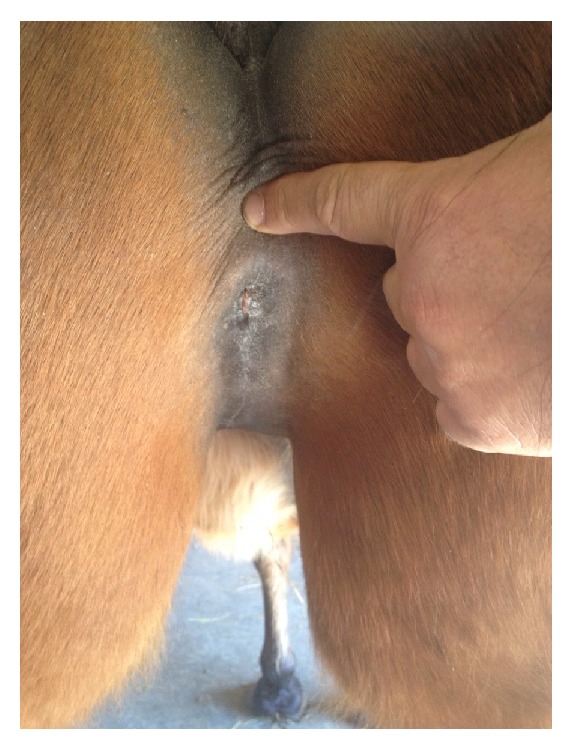
Macroscopic appearance of the perineal urinary meatus 3 years after surgery: urethral stoma underwent a progressive contraction until stabilization, allowing normal urination without urine scalding.

A 3-year follow-up showed no recurrence of the neoplasm with normal urination. Urethrostomy did not show any further stricture as well as no urine scalding (Figure 3).

## 3. Discussion

In this case report the long-term (3 years) successful treatment of a SCC of the penis body and prepuce in a gelding is described. The surgical treatment of the disorder was achieved with an* en bloc* extensive resection of penis and prepuce without penile retroversion as previously described [14] associated with a permanent perineal urethrostomy.

Differential diagnoses in presence of preputial masses, oedema, and discharge, difficulty at urinating, and phimosis are other neoplastic disorders (squamous papilloma, fibrosarcoma, adenocarcinoma, neurofibroma, basal cell carcinoma, and melanoma) [1, 2] and nonneoplastic pathologies such as epithelial hyperplasia, cutaneous infection with* Habronema* spp.,* Halicephalobus* spp., or* Draschia megastoma*, and coital exanthema by EHV-3 [2, 19, 20]. Although for antemortem diagnosis results of microscopic examination of cutaneous biopsy specimens or possibly aspirate samples of the lesion are useful [21] these were not undertaken because of the degree of swelling, abscessation, and ulceration at the penis and prepuce causing dysuria and systemic illness. Therefore a surgical approach was chosen to remove the mass and restore normal urinary function as a salvage procedure.

*En bloc* resection of penis and prepuce is indicated in cases where external genitalia are extensively affected by the neoplasia. It allows removal of the penis as far proximal as possible until healthy tissue is recognized. Despite invasiveness and postoperatory complications, this technique allows a high rate of success for the cases described in the literature with long-term survival and few tumour recurrences [1, 2, 4, 13, 15].

*En bloc* resection of penis and prepuce is usually associated with penile retroversion and suture of the penile stump to a subischial skin incision to create a new urinary meatus [13]. In the case series of Archer and Edwards the original technique of Markel was slightly modified to achieve an urethrostomy stoma in subischial position, about 20 cm below the anus, accomplished through a penile stump retroversion [15].

According to Van Harreveld et al. an option is to transect the penis just distal to the site typical of a routine perineal urethrostomy. The corpus cavernosum penis is closed and secured to local fascia and subcutaneous tissue, and a permanent perineal urethrostomy is performed. To our knowledge, no published reports of the long-term outcome of this surgical technique in the horse exist; the authors remark that this technique has worked well and appears to avoid the creation of a flexure in the penis [16].

Recently Wylie and Payne reported 15 cases of extensive penile disorders treated with a subischial urethrostomy and penile amputation with preputial ablation performed under general anaesthesia in dorsal recumbence. Eleven SCC, 2 melanomas, 1 chronic preputial discharge without neoplasia, and 1 paraphimosis secondary to sedation were included. They describe a median survival time of 25.1 months, with 64.3% (9/14 cases) surviving >18 months [18].

In the case described here, to find a normal macroscopic aspect of penile shaft and be consistently sure of the absence of neoplastic tissue left, the penile amputation had to be made very close to the ischiatic arch. To remove all the affected tissue the penile body was excised extensively, more proximally than in the position described by Archer and Edwards [15]; for this reason not enough penile tissue was left to perform a retroversion. So the urinary flow was diverted through a permanent perineal urethrostomy. Differently from the paper of Wylie and Payne, a permanent urinary meatus was created in the perineum after penile amputation, as distal as possible from the anus to avoid faecal contamination and urine scalding. Performing penile amputation before urethrostomy allows the surgeon to evaluate intraoperatively the degree of infiltration of the penile body giving the opportunity to adjust the extension of the amputation.

Although penile retroversion allows the surgeon to direct urinary outflow more caudally than does penile amputation without retroversion [13], no problems due to dysuria or misdirection of the urinary flow and consequent urine scalding were encountered.

In order to reduce ascending infections from the external urinary meatus, the ventral retractor penis muscles were sutured to the subcutis at the urethrostomy site, hypothesizing that their contraction could act as an external urinary sphincter closing the external opening of the stoma at the end of urination, avoiding bacteria ingress, urine dripping, and secondary scalding. The suture of the muscles to the subcutis ensured also a secure fixation of the penis stump, avoiding subcutaneous infiltration of urine.

The complications after surgery encountered in this case were not different from those already described, particularly haemorrhage from the corpus spongiosum at the end of urination, infection of the abdominal wound, and partial dehiscence of the urethral mucosa sutured to the perineum [13, 22, 23].

Postoperative haemorrhage was managed with the administration of tranexamic acid until bleeding stopped. Although haemorrhage is a well described major complication associated with this surgery, the preemptive use of antifibrinolytic agents was not attempted because of the concern about a possible increase in the risk of thromboembolic complications, such as deep vein thrombosis and acute myocardial infarction, related to the use of these drugs in human medicine [24, 25]. Furthermore there are only few refereed studies validating the use of these drugs in the horse. Failure of response to procoagulant agents could have been managed through blood transfusion.

Infection of the abdominal wound was managed by local daily disinfection and use of antimicrobial without other sequelae.

The dehiscence of a permanent urethrostomy can be due to the excessive manipulation of urethral tissue [26] or to excessive tension on the sutures; in our case the haematic infiltration of the subcutaneous tissue consequent to the bleeding from the corpus spongiosum could have increased tension on the urethrostomy sutures leading to a partial dehiscence.

Even though stricture formation can be a complication in 1/3 of perineal urethrostomy cases during early postoperative period and after removal of the urinary catheter [23–26], we did not observe dysuria due to urethral stricture. Two months after surgery a relative stenosis of the urethrostomy occurred, not affecting urinary emission.

The advantages of the* en bloc* extensive resection of the penis and prepuce without penile retroversion associated with a permanent urethrostomy were the removal of as much penile body as possible, minimizing recurrence and avoiding tension related to phallopexy, and the possibility to create a urinary meatus which is functional and cosmetically similar to the mare's.

Postoperative histopathology revealed an infiltration of neoplastic cells in the subcutaneous tissue close to the albuginea, but not in the* corpus cavernosum penis*, which was affected by chronic balanitis, worsened by the presence of myiasis; the chronic balanitis probably contributed to the palpable thickening of the body of the penis, simulating neoplastic infiltration. Although macroscopically enlarged, inguinal lymph nodes did not contain neoplastic cell, but, consistently with literature [1], only marked signs of regional inflammation and lymphoid hyperplasia. Whenever phlegmon and inflammation of the inguinal tissues are present, the sensibility and specificity of ultrasonographic examination and fine needle aspiration of inguinal lymph nodes are reduced [1]. So in order to avoid recurrences extensive surgery was recommended.

The lack of a standardized approach to follow-up evaluation and the definition of clinical success limit an objective consideration about the efficacy of the treatment itself and its prognostic value. Since most of the surgical treatments for penile and preputial disorders are considered as salvage procedures, compromising reproductive and original anatomy, it is essential to the clinician to perform a detailed evaluation of each clinical case and the appropriate treatment.

In this case report the* en bloc* extensive resection of the penis and prepuce without penile retroversion and permanent urethrostomy allowed the successful treatment of a locally invasive SCC of penis and prepuce in a gelding without recurrence after a 3-year follow-up time. Although short- and long-term complications occurred, they were managed and did not compromise the clinical outcome.
